# Differentiation of human bone marrow stem cells into cells with a neural phenotype: diverse effects of two specific treatments

**DOI:** 10.1186/1471-2202-7-14

**Published:** 2006-02-16

**Authors:** Franca Scintu, Camilla Reali, Rita Pillai, Manuela Badiali, Maria Adele Sanna, Francesca Argiolu, Maria Serafina Ristaldi, Valeria Sogos

**Affiliations:** 1 Department of Cytomorphology, University of Cagliari, Italy; 2 Bone Marrow Transplantation Unit, Ospedale Regionale per le Microcitemie, Cagliari, Italy; 3 Department of Biomedical Science and Biotechnology, University of Cagliari, Italy; 4 INN, CNR, Cagliari, Italy

## Abstract

**Background:**

It has recently been demonstrated that the fate of adult cells is not restricted to their tissues of origin. In particular, it has been shown that bone marrow stem cells can give rise to cells of different tissues, including neural cells, hepatocytes and myocytes, expanding their differentiation potential.

**Results:**

In order to identify factors able to lead differentiation of stem cells towards cells of neural lineage, we isolated stromal cells from human adult bone marrow (BMSC). Cells were treated with: (1) TPA, forskolin, IBMX, FGF-1 or (2) retinoic acid and 2-mercaptoethanol (BME). Treatment (1) induced differentiation into neuron-like cells within 24 hours, while a longer treatment was required when using retinoic acid and BME. Morphological modifications were more dramatic after treatment (1) compared with treatment (2). In BMSC both treatments induced the expression of neural markers such as NF, GFAP, TUJ-1 and neuron-specific enolase. Moreover, the transcription factor Hes1 increased after both treatments.

**Conclusion:**

Our study may contribute towards the identification of mechanisms involved in the differentiation of stem cells towards cells of neural lineage.

## Background

Up until only a few years ago, the fate of adult stem cells was believed to be limited to their tissue of origin. However, it has recently been demonstrated that in several tissues adult stem cells may undergo a fate other than that habitually manifested in physiological conditions. As an example, stem cells isolated from bone marrow can differentiate not only into blood cells, but also into hepatocytes [1], skeletal muscle [2] and cardiomyocytes [3]. Even more surprisingly, recent studies have shown that stem cells in vivo may even give rise to neural cells, thus representing an important alternative to embryonic cells for therapeutic transplantation [4,5]. Indeed, the original idea of substituting neurons damaged by different pathological events with undamaged heterologous neurons has not so far afforded satisfactory results [6,7]. On the other hand, although the discovery of the existence of neural stem cells in the adult human brain [for a review see [8]] is certainly a milestone in the field, it is difficult to conceive a method leading to manipulation and reimplantation of neural stem cells taken directly from the patient's brain. Accordingly, the source represented by bone marrow offers theoretically unlimited therapeutical applications.

Two major subpopulations of stem cells can be identified in bone marrow: hematopoietic stem cells, which give rise to red blood cells, platelets, monocytes, granulocytes and lymphocytes, and non-hematopoietic stromal cells (BMSC), more recently acknowledged to be of a mesenchymal nature due to their capacity to differentiate into myogenic, osteogenic, chondrogenic and adipogenic lineages [9,10]. BMSC can be isolated from whole bone marrow by adherence to plastic culture dishes. Recent studies have shown that BMSC can be induced to express a neuronal phenotype in vitro under specific experimental conditions. For example, Woodbury et al. [11] observed that in the presence of β-mercaptoethanol and dimethylsulfoxide BMSC may differentiate into cells expressing neuron specific enolase (NSE) and neurofilament. Moreover, it has been described that in the presence of epidermal growth factor (EGF) and brain-derived neurotrophic factor (BDNF) stromal cells differentiate into neural cells that express both neuronal and glial markers (Neu-N, nestin, GFAP) [12]. Deng et al. [13] demonstrated how factors that increase intracellular cAMP induce the differentiation of BMSC into early progenitors of neural cells. Unfortunately, due to the differences in BMSC isolation and culture conditions, as well as in characterization of differentiated cells, it is not possible to compare these treatments. For this reason, we examined the different effects produced by two specific treatments in inducing differentiation of BMSC into neural phenotype. Recent studies show that the transcription factors with helix-loop helix (bHLH) motifs [for review see: [14,15]] are essential for maintenance of neural precursor cells in an undifferentiated state. bHLH genes include the mammalian Hairy and Enhancer of Split genes (Hes1, Hes3 and Hes5); in particular Hes1 is highly expressed by neural stem cells [15,16]. Our results show that Hes1 is involved in BMSC differentiation into neural cells. In fact a transient increase of this transcription factor was observed in neural precursors after both treatments.

## Results

### BMSC characterization

**Figure 1:**
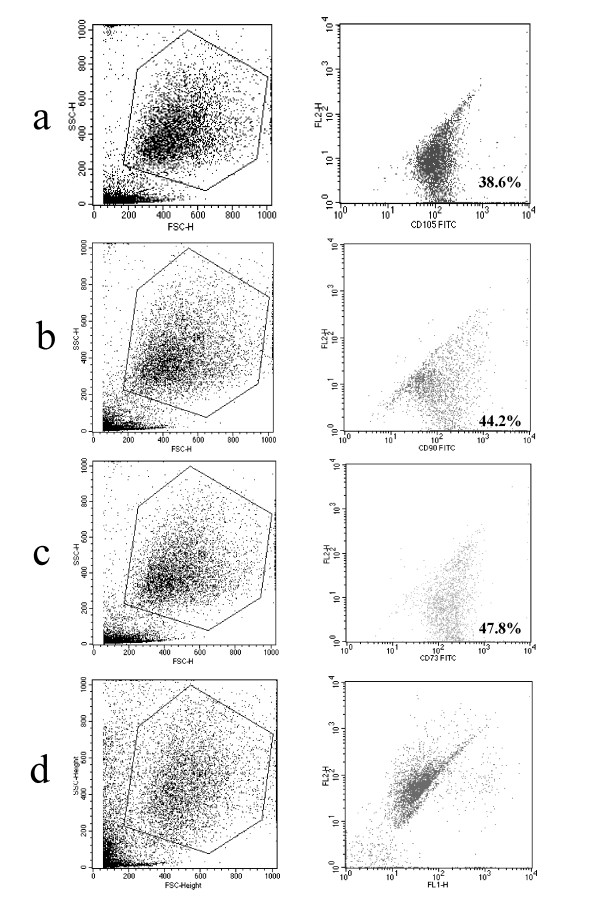
FACS analysis of BMSC. The percentage of positive cells for CD105 (a), CD90 (b) and CD73 (c) is indicated in the lower right-hand corner of each graph. (d) negative control.

BMSC were isolated from human bone marrow aspirates and propagated in culture. To characterize these cells, fluorescent cell sorting was performed using antibodies against mesenchymal cell surface markers, such as CD90, CD105 and CD73. Cells were positive for all these markers, showing a typical mesenchymal-like immunophenotype (fig. 1). On the other hand they were negative for CD34, CD11b and CD13, markers associated with hematopoietic cells (data not shown).

### Induction of neural phenotype

#### Cellular morphology

**Figure 2:**
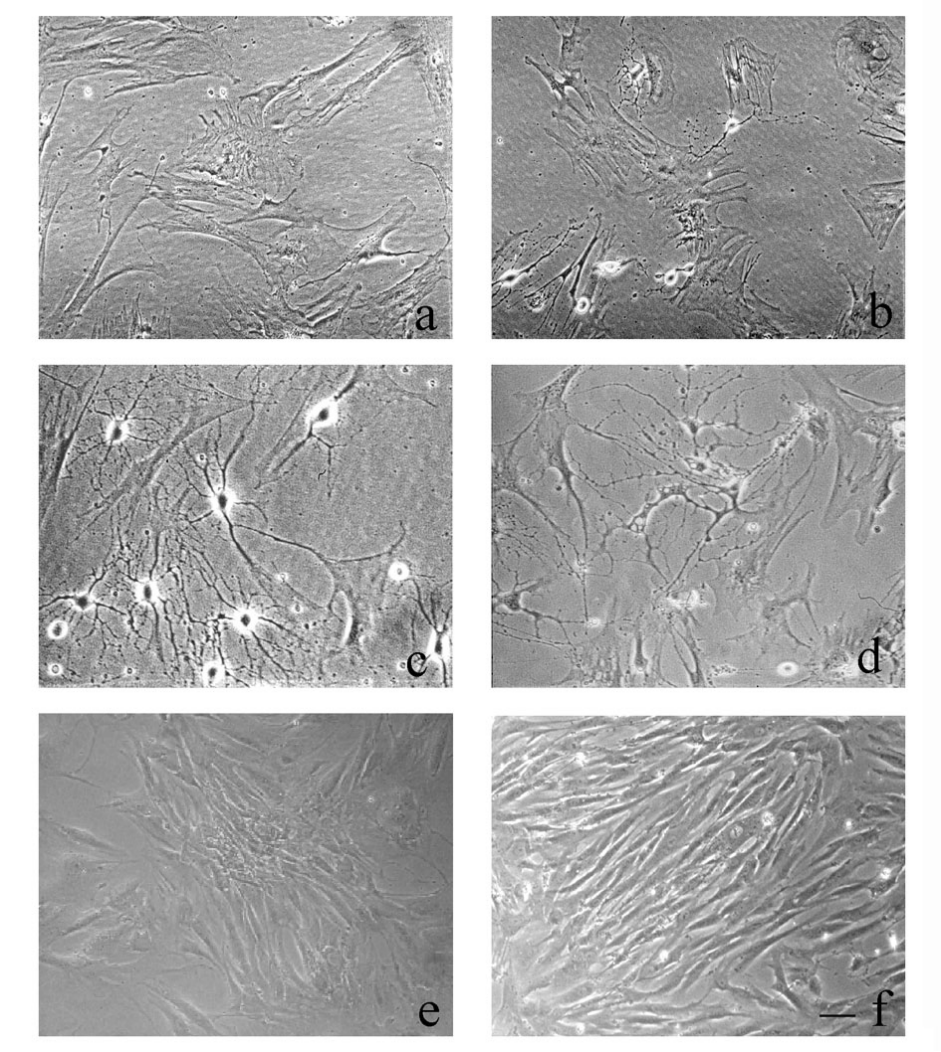
Phase-contrast photomicrograph of BMSC and those exposed to treatment 1 or to treatment 2 for 3 (e) and 7 days (f). In untreated cultures (a), BMSC showed a large, flat morphology. After 5 hours (b) of treatment 1 and even more after 24 hours (c), cells had morphological features typical of neurons, such as spherical shape and extending processes; this morphology also persisted at 48 hours of treatment (d). The treatment 2 determined, after 3 days, an increase of cell density (e). After 7 days (f) cells with a spindle-shaped morphology could be observed. Bar: 80 μm.

**Figure 3:**
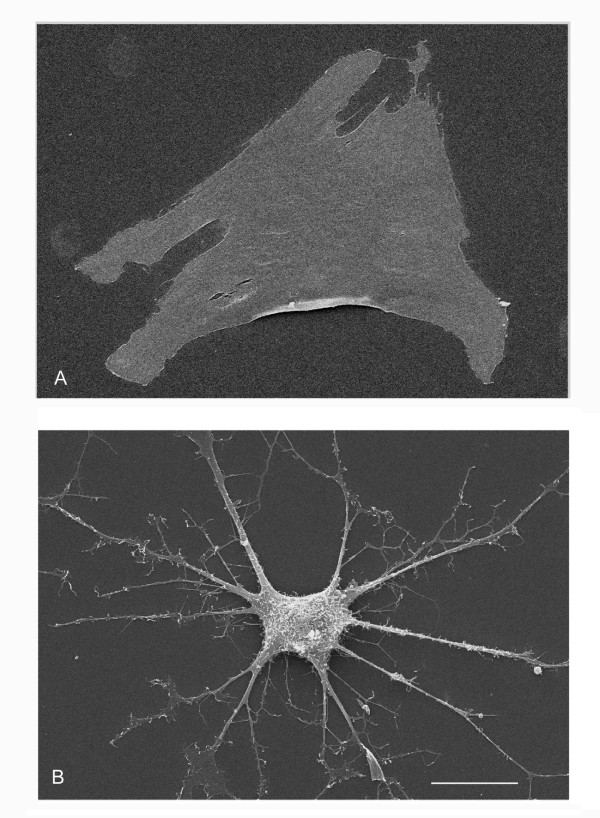
Scanning electron microscopy (SEM) of untreated BMSC (A) and after 24 h of treatment 1 (B). Bar: 20 μm (B).

Undifferentiated BMSC displayed a flat morphology with short processes (fig. 2a). To identify conditions that may induce differentiation towards a neural phenotype, two different in vitro treatment protocols were used. When cells were treated with treatment 1, a cocktail of fibroblast growth factor-1 (FGF-1) and co-activating substances such as TPA (protein kinase C (PKC) activator), IBMX and forskolin (protein kinase A (PKA) activator), their morphology, observed by contrast phase and scanning electron microscopy, changed rapidly. Indeed, after 5 hours (fig. 2b), cytoplasm was observed to have retracted toward the nucleus in some cells, taking on a more spherical shape and extending processes. The percentage of cells modifying morphology increased progressively up to 24 hours (fig. 2c). Subsequently, proliferation in these cells came to a halt and after 48 hours (fig. 2d) of treatment a senescent morphology was evidenced. These modifications were more evident at scanning electron microscopy (fig. 3). When differentiation factors were removed from medium, cell morphology reverted to the original BMSC shape (data not shown). On the contrary, treatment 2 (retinoic acid and BME) did not determine similarly rapid and dramatic changes: as shown in fig. 2e, an increase of cell density with consequent morphological modifications could be observed only after 3 days. After 7 days (fig. 2f) the majority of cells were seen to have assumed a spindle-shaped morphology which persisted for up to 10 days following treatment (data not shown).

#### RT-PCR

**Figure 4:**
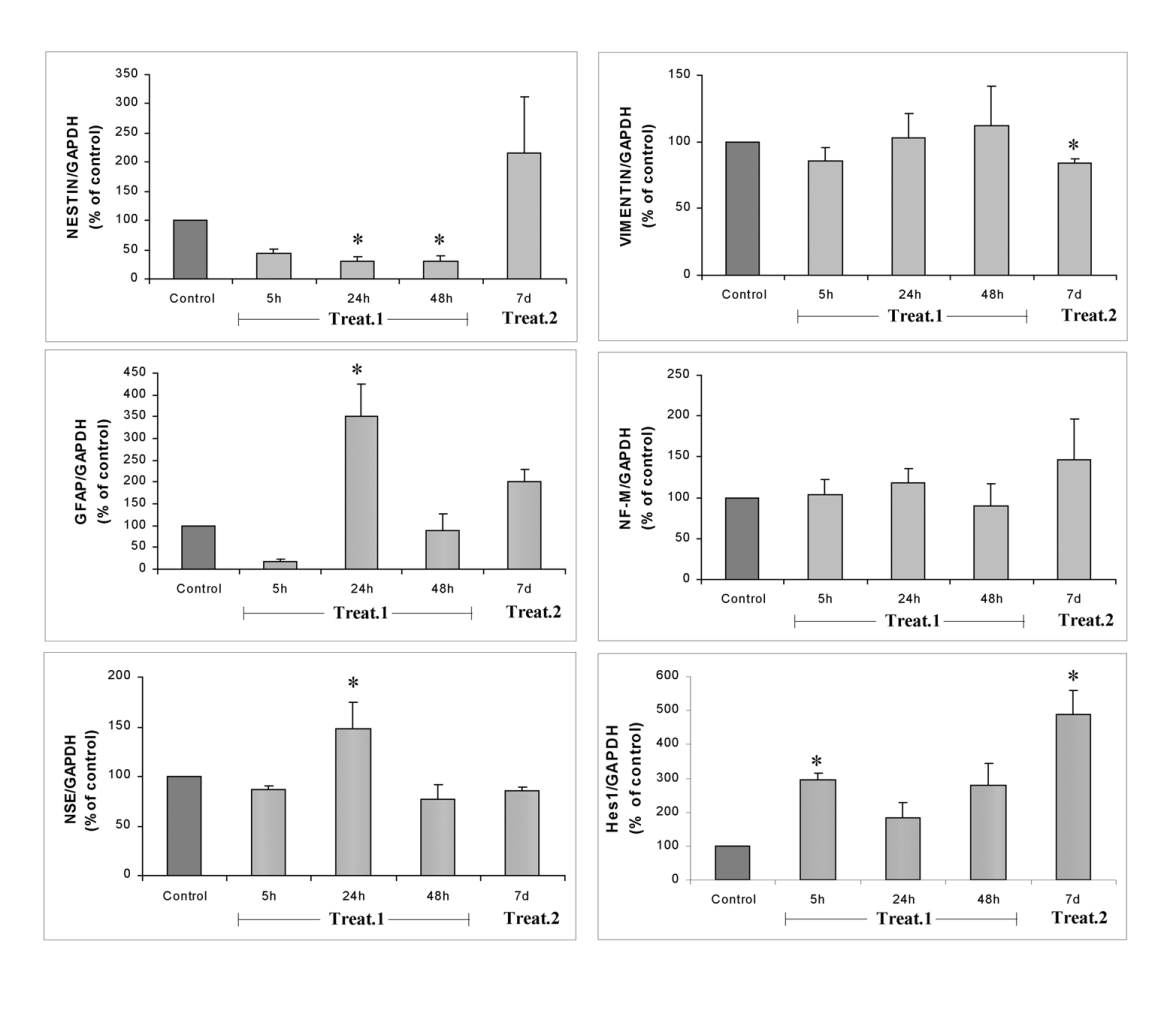
RT-PCR for neural markers in BMSC after normalization to GADPH. Treatment 1 induced a significant decrease in nestin mRNA and an increase in GFAP and NSE expression after 24 h of treatment; Hes1 expression increased after 5 h and it was reduced during differentiation (24 h, 48 h). Vimentin and neurofilament mRNA levels were unchanged. On the contrary, after 7 days of treatment 2 only vimentin mRNA expression significantly decreased. Moreover, treatment 2 determined a significant increase in Hes1 mRNA expression after 7 days; no change was observed in other mRNA levels respect to controls. *P < 0.05 vs. control, (n = 4).

The expression of neural genes in both undifferentiated and differentiated BMSC was examined in order to verify the occurrence of neural differentiation after treatment. Total mRNA was extracted from untreated and treated cells and analyzed by RT-PCR (fig. 4). Nestin mRNA was present in undifferentiated BMSC, although expression was observed to have decreased in a significant and progressive manner at 24 and 48 hours after treatment 1. GFAP and NSE were weakly expressed prior to both treatments, although treatment 1 determined for both proteins a significant increase at 24 hours and then subsequently a decrease. No significant differences in vimentin and neurofilament mRNA levels were revealed by RT-PCR analysis between controls and treated cells after treatment 1. When BMSC were treated with retinoic acid, no change was observed in nestin mRNA levels respect to controls. However, retinoic acid led to a significant decrease of vimentin, while GFAP, NF and NSE mRNA levels were not modified.

RT-PCR for Hes1 was performed in untreated and treated BMSC. The graph in fig. 4 shows that Hes1 was expressed at low levels in undifferentiated BMSC, but its mRNA levels significantly increased after 5 hours of treatment1. Hes1 expression tended to decrease as neural differentiation proceeds. In fact, after 24 h and 48 h of treatment 1, mRNA levels were not significantly different as compared to undifferentiated cells. At the same manner, treatment 2 determined a significant increase of Hes1 expression after 7 days.

#### Western blot

**Figure 5:**
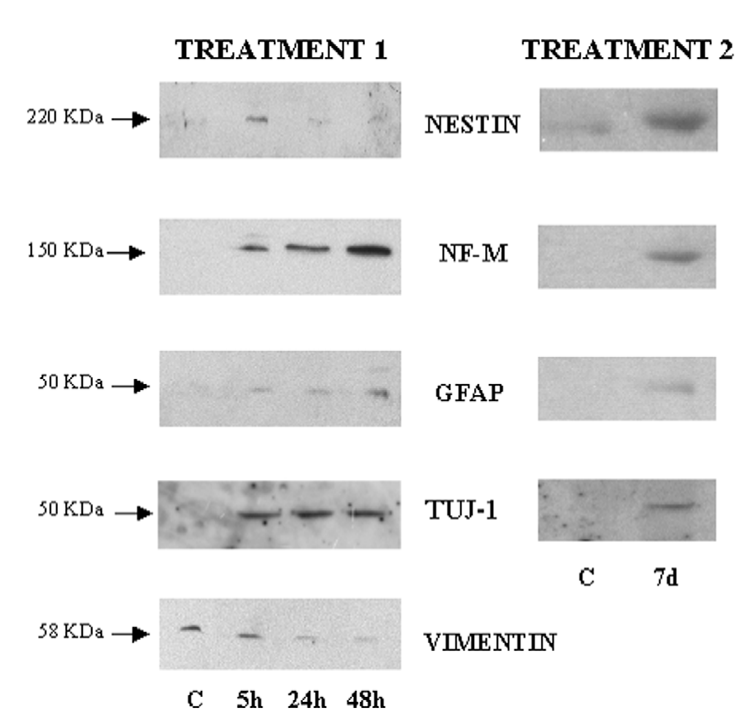
Western blot analysis of neural markers after treatment 1 or 2 of BMSC. The results showed that both treatments produced an increase of neural markers respect to untreated cells. On the contrary, vimentin, well expressed in untreated BMSC, decreased from 5 to 48 hours of treatment 1.

Western blot analysis of cultures revealed how both treatments had modified the expression of specific neural markers. As shown in fig. 5, untreated BMSC were positive for vimentin and displayed a weak expression of nestin, a marker for neural precursor. Cells were negative for other neural markers, such as NF, GFAP and TUJ-1. A strong expression of nestin was observed in treated cells at five hours from treatment 1, which was subsequently followed by a decrease in levels of this protein. Treatment 1 also induced expression of neurofilaments, which were seen to be present after 5 hours and to have further increased at 48 hours. On observation at 5 hours from treatment 1 GFAP was already present, with a progressive increase having occurred at 24 and 48 hours. Moreover, a strong expression of TUJ-1 appeared after 5 hours and was maintained until 48 hours from treatment 1. However, an opposite trend was displayed by vimentin which decreased from control to 48 hours. Following treatment with retinoic acid, vimentin was negative (data not shown), although after 7 days this treatment produced an increase in nestin, neurofilaments, GFAP and TUJ-1.

#### Immunocytochemistry

**Figure 6:**
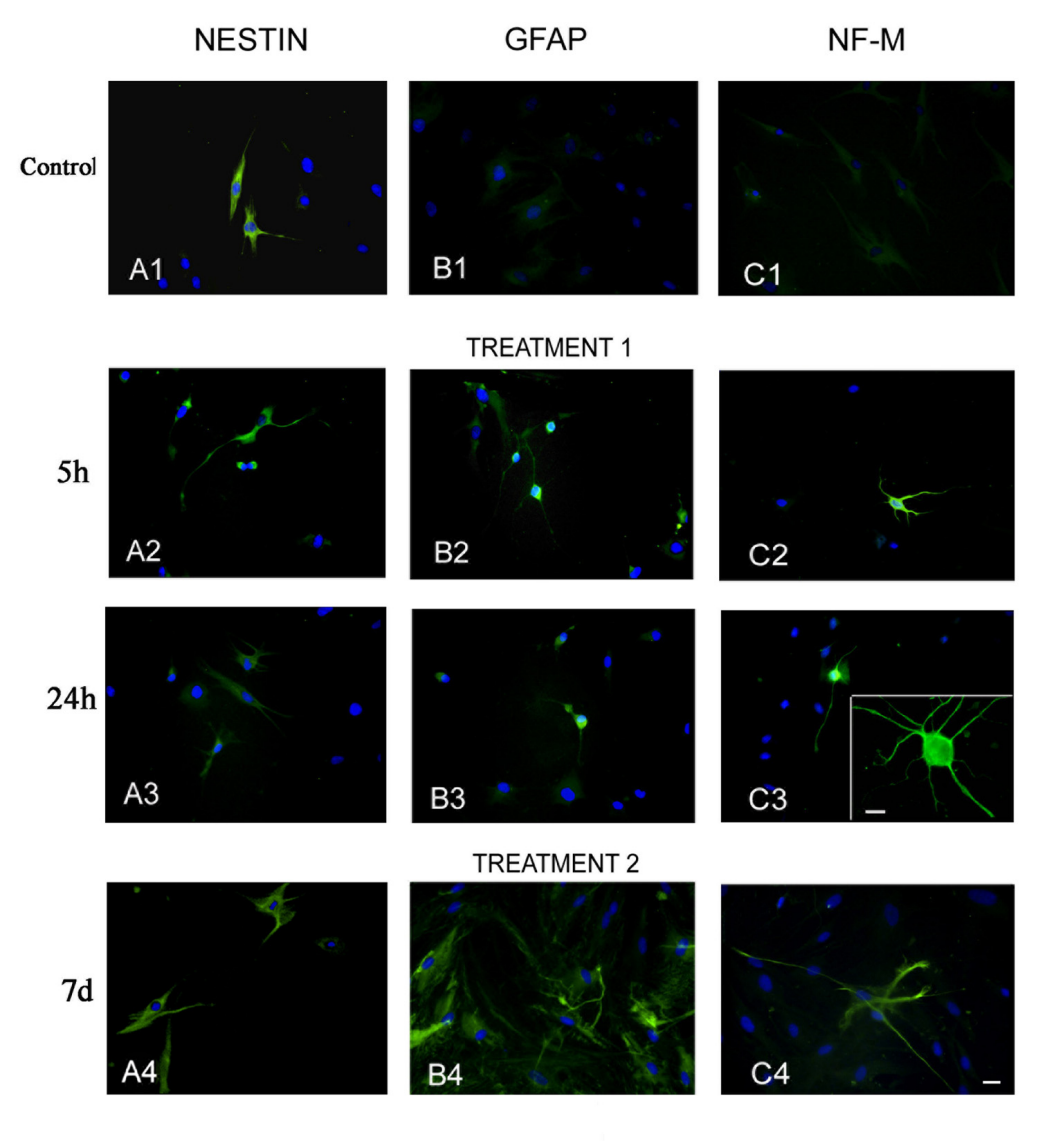
Expression of nestin (A), GFAP (B) and neurofilament (C) in undifferentiated and differentiated BMSC. Untreated cells were labeled for nestin (A1), whose expression decreased with treatment 1 (A2,3) but persisted after treatment 2 (A4). GFAP immunoreactivity was negative in untreated cells (B1), but after 5 (B2) and 24 hours (B3) of treatment 1 some cells were GFAP positive. Treatment 2 determined strong GFAP immunostaining after 7 days (B4). Immunoreactivity for neurofilaments, totally absent in untreated BMSC (C1), was present in some cell bodies and processes after treatment 1 (C2,3; higher magnification in C3 insert) and treatment 2 (C4). Cells were also stained with Hoechst dye 33258. Bar: 40 μm. Insert bar: 10 μm.

**Figure 7:**
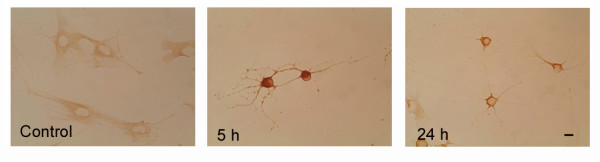
Immunocytochemical staining of BMSC for NSE after 5 and 24 hours of treatment 1. Treated cells expressed high levels of NSE compared to those untreated. Bar: 20 μm.

Immunocytochemistry was performed to investigate cellular localization of neuronal markers. Analysis confirmed that immunoreactivity for specific neural markers had been induced by both treatments (fig. 6). Nestin was present in untreated BMSC (A1) and had increased 5 hours after treatment 1 (A2). On the contrary, by means of cell differentiation (24–48 hours), a decrease of staining intensity was registered for the expression of nestin (A3,4). GFAP immunoreactivity, absent in untreated cells (B1), was manifested 5 hours after treatment 1 and persisted until 24 hours after treatment (B2,3). Immunostaining for neurofilaments was totally absent in untreated BMSC (C1), although after treatment 1 immunoreactivity was visible in some cell bodies and processes (C2,3), more evident at higher magnification (C3 insert). Neuron-specific enolase expression (fig. 7) had already been induced by PK activators 5 hours after treatment and persisted after 24 hours. Treatment 2 determined strong GFAP (B4) and neurofilament (C4) immunostaining in several cells after 7 days. On the other hand, nestin immunostaining was not modified by this treatment (A4).

## Discussion

Several recent studies have suggested that stromal cells from bone marrow may be capable of generating either neurons or glia, both in vivo [4,5] and in vitro [12].

In this study we induced stromal cells from human bone marrow to differentiate into cells with a neural phenotype, comparing two different protocols. Furthermore, we explored for the first time the involvement of the transcription factor Hes1 in inducing neural phenotype in BMSC. Both treatments led to a neural phenotype in a similar but not identical manner. Indeed, at the end of both treatments, BMSC expressed neural markers such as NF, TUJ-1 and GFAP, whereas nestin and vimentin progressively decreased with differentiation. These results were associated with different morphological modifications and required times of exposure ranging from one day to one week, depending on the treatment. The first induction media (a cocktail of PKC and PKA activators and FGF-1) rapidly determined dramatic modifications of cell shape and induced the expression of several neural markers in less than 24 hours. Differentiation was transient and cells reverted to their original phenotype when the inducing factors were removed. On treating BMSC with retinoic acid and BME biochemical markers consistent with differentiation towards a neural phenotype were not expressed before seven days. Moreover, morphological changes were not as drastic as in the other treatment, although differentiation persisted even when inducing agents were removed.

It is well known that an increase of intracellular cAMP and consequent PKA activation represents a critical regulator for differentiation of neurons and glia [17]. Moreover, agents that increase intracellular cAMP levels induce neuroendocrine differentiation in human prostate carcinoma cells [18], neuronal differentiation in C6 glioma cells [19,20] and processes elongation in MCD-1 medulloblastoma cell line [21]. In the same manner, PKC is involved in neural differentiation regulating neurite outgrowth and branching [22,23]. Iacovitti et al. [24] showed that FGF-1, together with co-activator molecules such as dopamine, PKA and PKC activators, induced differentiation of an embryonic carcinomal stem cell line into dopamine neurons. Deng et al. [13] found that human BMSC can be differentiated into early neural progenitors by conditions that increase intracellular cAMP. After six days of treatment these cells expressed NSE and vimentin but not markers of mature neurons, such as NF-M or MAP-2. In our experiments, treatment 1 induced BMSC to differentiate into more mature neuron-like cells within 24 hours.

Retinoic acid (RA), a derivative of vitamin A, is essential in maintaining normal cellular growth and development. In fact, RA is present in various tissues of both embryonic and adult animals, in particular in the nervous system [25-30], where it promotes neuronal differentiation [31]. Previous studies have demonstrated that RA induces both a greater number of neurites as well as increased neurite length in cultured neurons [for a review, see [32]]. Retinoic acid has been used in combination with other factors to induce differentiation of BMSC into neural cells [12,33-35]. Since it has been suggested that BME is capable of supporting the viability and differentiation of fetal mouse brain neurons [36], we used low concentrations of this factor in combination with retinoic acid (treatment 2). With this treatment, BMSC slowly differentiated into neuron-like cells and after 7 days they expressed NF-M. Cells survived in induction media for up to at least 14 days.

These results confirmed data reported by other authors suggesting that BMSC are able to differentiate in cell types of varying embryonic origin. In particular, using two different treatments we induced BMSC to differentiate into cells with neuronal phenotype. At the end of both treatments cells expressing neural markers such as neurofilament and GFAP were obtained, whereas vimentin and nestin decreased with differentiation. The presence of GFAP in treated BMSC may be ascribed not only to differentiation into astrocytes, but also to neural precursors, that have been demonstrated to express this protein [37].

Prior to inducing differentiation, BMSC expressed nestin, a commonly used marker of neural precursor. A transient expression of this intermediate filament in non-neural cells, such as hepatic stellate cells, myogenic and epithelial cells has recently been observed [38,39]. Moreover, in our experiments the patterns of mRNA and protein expression did not exactly match. For example, mRNA for neurofilaments was present in BMSC and was not modified during differentiation, whereas the protein was absent in untreated cells and its expression was induced by treatments. These data suggest that the expression of neural markers (such as neurofilaments, nestin, GFAP, NSE) is controlled at a translational rather than a transcriptional level. In fact, the constitutive expression of these proteins by BMSC confirms the hypothesis that these cells are "multidifferentiated" cells and thus can retain the ability for neuronal differentiation, as already suggested by Tondreau et al. [40].

Microscopic examination of the cultures revealed that, using either of the two protocols, only a small percentage of cells (15–20%) showed immunoreactivity for neural markers, suggesting that only a subset of BMSC can differentiate into cells with a neural phenotype. We can therefore hypothesize that this subfraction of BMSC may be constituted by tissue-specific progenitor cells with restricted differentiation potential, capable of giving rise, under specific experimental conditions, to cells characteristically found in other tissues. In fact, morphological and phenotypic heterogeneity of BMSC has been demonstrated: BMSC include small round cells and large polygonal-shaped cells with different multilineage potentials [41,42]. On the other hand, our findings may indicate the possibility that a population of multipotential stem cells resides in adult bone marrow.

The results reported demonstrate how the microenvironment may be capable of affecting cellular differentiation, since activation of PKs alone leads to neuron-like cell morphology, but the cells express neural markers after both treatment protocols. This observation suggests that specific marker expression is not always accompanied by typical morphological modifications [5,43]. In contrast, Black and Woodbury [44] have demonstrated that BME induces stromal rat cells to differentiate into neuronal phenotype cells, but at concentrations 10–100 times higher than those used in the present study. In our experiments, RA and BME concentrations were sufficient to affect the expression of neural markers but not to induce morphological modifications. The differences observed between the two treatments respect to morphological changes and to time required for differentiation may indicate the activation of different intracellular molecular mechanisms in these processes. It has been suggested that the molecular mechanisms involved in the action of RA during embryogenesis may occur via a complex signaling pathway [for review see [45]]; moreover, it has been hypothesized that both in vitro and in vivo, the number of neural-specific genes encoding transcription factors and signaling molecules induced by RA during neural differentiation are comparable [46].

In a recent report Lu et al. [47] observed a neuron-like morphology in BMSC exposed to various stressors. An increase in NSE immunoreactivity was also observed, but it could not be confirmed by RT-PCR, suggesting that these modifications can be the result of cell shrinkage. On the contrary, our results showed the increase of neural markers not only by immunocytochemistry, but also by RT-PCR and western blot.

Hes1 is a transcription factor that plays a key role in neurogenesis regulation maintaining cortical progenitors by inhibiting neurogenesis [15]. Our results show that after 5 hours of treatment 1, the expression of Hes1 transiently increases. We could speculate that the upregulation of Hes1 expression induces differentiation of BMSC into neural precursors and its inhibition leads these cells to differentiate into more mature cells, as observed in vivo during development [16]. On the other hand, we observed an increase of Hes1 expression after 7 days of treatment 2. This result may indicate that retinoic acid treatment leads cells to acquire a less mature phenotype as compared to treatment 1. This hypothesis is confirmed by nestin expression that decreases after treatment 1, but that is not modified by treatment 2.

## Conclusion

As summarized in the present report, it has been demonstrated that different differentiation-inducing factors and protocols are capable of generating neural cell types from BMSC. Moreover, we observed a modulation of Hes1 expression with both treatment, suggesting an involvement of this transcription factor in differentiation of BMSC into neural cells. By combining these molecular biological strategies and neuronal differentiation systems, it should prove possible to detect, isolate, and characterize specifically expressed genes involved in neuronal differentiation. Our results suggest that adult bone marrow stem cells may differentiate into cells with distinct glial or neuronal phenotypes after exposure to specific signals or modulating specific genes, such as Hes1, and thus constitute a cellular reservoir for neurodegenerative diseases of the central nervous system.

## Methods

### Cell culture

20–40 ml bone marrow aspirates were taken from 10 healthy donors (age range 6–33 years) after obtaining of informed consent. Mononuclear cells were separated by gradient density using Ficoll-Hypaque and MSC selected by plastic adhesion according to Lee et al. [48]. Briefly, cells were seeded in culture flasks including α-MEM (Cambrex) supplemented with 20% FCS and 10^-8^M vitamin K. After 24 hours, 10^-8^M dexamethasone and 50 μg/ml ascorbic acid were added without media change. Subsequently, the culture flasks were left undisturbed for 4–5 days to promote cell attachment. After this time, the non-adherent cells were removed by replacing the medium. At near-confluence, cells were subcultured after trypsin digestion.

### Cell treatment

After at least four passages, cells were plated at a density of 2 × 10^4 ^cells/ml in a 10-cm plastic culture dish for western blot and RT-PCR, and in 3.5-cm dishes containing glass coverslips for immunocytochemistry and electron microscopy. After 24 hours, cells were treated with two different protocols:

#### treatment 1

10 ng/ml FGF-1 (R&D, Minneapolis, MN), 200 nM 12-O-tetradecanoylphorbol-13-acetate (TPA; Sigma, St Louis, USA), 250 μM IBMX (Sigma) and 50 μM forskolin (Sigma), in DMEM/F12 (Sigma) 1:1 supplemented with ITS (Sigma) 1%.

#### treatment 2

1 μM all-trans-retinoic acid (Sigma) and 100 μM 2-mercaptoethanol in DMEM supplemented with 10% FCS (Invitrogen).

At various time intervals, cultures were fixed for immunocytochemistry and electron microscopy, or processed for RT-PCR and western blot.

#### Immunocytochemistry

Cells on coverslips were fixed at -20°C with cold methanol for 4 min. For staining, samples were rehydrated in PBS/0.2% (v/v) Triton X-100 and pre-incubated with normal goat serum (1:5, Vector, Burlingame, CA, USA) for 30 min. Cells were then overlaid with the following primary antibodies diluted in PBS/0.2% (v/v) Triton, for 60 min at room temperature: monoclonal anti-nestin (1:100, Chemicon, Temecula, CA, USA); polyclonal anti-GFAP (1:200, DAKO, Carpinteria, CA, USA); polyclonal anti-neurofilament 150 kDa (1:100, Chemicon). Texas red conjugated anti-mouse IgG (1:200; Jackson ImmunoResearch, West Grove, PA, USA) or FITC conjugated anti-rabbit IgG (1:500; Molecular Probes, Eugene, OR, USA) were used as secondary antibodies. After several washings samples were overlaid with Hoechst 33258. Negative controls were incubated with non immune serum and with appropriate secondary antibody.

In other experiments, cells were incubated with 1% phenylhydrazine hydrochloride (Sigma) to block endogenous peroxidase for 30 min and then with monoclonal antibodies against neuron specific enolase (NSE; 1:20, Cymbus Biotechnology) for 60 min at room temperature. Subsequently, slides were rinsed three times in PBS-Triton, incubated for 30 min with biotinylated antimouse IgG (1:200, Vector), rinsed and incubated with avidin D-conjugated horseradish peroxidase (1:800, Vector) followed by 10 min incubation with DAB tablet sets (SIGMA FAST). Negative controls were incubated with non immune serum instead of primary antibody.

### Western Blot

Cells were lysed with sodium dodecyl sulphate (SDS) 2%. Protein concentration was measured according to the method of Lowry [49]. Loading buffer (75 mM Tris-HCl, pH 6.8, 20 % glycerol, 5% 2-mercaptoethanol, 0.001 bromophenol blue) was subsequently added and samples were placed in boiling water for 3 min. 15–30 μg of protein were run on 10% or 5% SDS-polyacrilamide gel, depending on molecular weight. Separated proteins were electroblotted onto PVDF membrane (Hybond-P, Amersham) and blocked with 5% nonfat dry milk overnight at 4°C. Immunodetection was performed with antibodies against nestin (1:1000, monoclonal, Chemicon, Temecula, CA, USA), GFAP (1:5000, polyclonal, DAKO, Carpinteria, CA, USA), neurofilament (1:5000, polyclonal, Chemicon), vimentin (1:1000, monoclonal, DAKO, Carpinteria, CA, USA), NSE (1:5000, monoclonal, Cymbus Biotechnology), TUJ-1 (β-III tubulin; 1:500, monoclonal, Chemicon) and GAPDH (1:600, monoclonal, Chemicon). The membrane was washed and incubated with a horseradish peroxidase conjugated anti-mouse IgG (1:1000, Chemicon) or anti-rabbit IgG (1:5000, Chemicon). After washing, protein bands were detected with SuperSignal West Pico chemiluminescent substrate (Pierce).

### Reverse transcription-polymerase chain reaction (RT-PCR)

Total RNA was isolated from cell cultures using Trizol reagent (Gibco) according to the method of Chomczynski [50]. The RNA concentration was measured in a spectrophotometer at 260 nm. Identical amounts of RNA were reverse transcribed into cDNA. cDNA was subsequently amplified by the polymerase chain reaction (PCR) with specific primers (Table 1). To obtain quantitative PCR with a non radioactive label, digoxigenin-11-dUTP (DIG) (Roche) was incorporated during PCR reaction. An aliquot of the PCR reaction mixture was electrophoresed in 3% agarose gels in Tris-borate/EDTA buffer to allow adequate separation of the cDNA. cDNA was transferred to a nylon membrane by blotting for 16 h in 10 × SSC. The blot was washed briefly in buffer A (maleic acid, 0.1 M; sodium chloride 0.15 M, pH 7.5, plus Tween 20, 0.3%) and incubated for 30 min at room temperature in blocking buffer (1% blocking reagent, Roche, in buffer A). This was followed by a 30 min incubation period with an antidigoxigenin-IgG conjugated to alkaline phosphatase (Roche) diluted in blocking buffer. The blot was washed and chemiluminescent detection performed by incubating the membrane for 5 min in a CSPD solution (Roche). The membrane was sealed in a clear plastic bag and exposed to X-ray film (KODAK X-OMAT) 2–10 min in an X ray cassette at room temperature. Signals corresponding to each molecule of interest and GAPDH mRNA were scanned using a densitometer (Hoefer Scientific Instruments, San Francisco, CA, U.S.A.). mRNA amounts were normalized by comparison with GAPDH levels. Differences in mRNA amounts between control and treated cells were evaluated using Student's t test for paired samples.

### Fluorescence-activated cell sorting (FACS)

For surface protein expression, BMSC were detached with 0.5% trypsin for 10–15 min. at 37°C, washed, counted and resuspended in 0.1% BSA/PBS and incubated with the following primary antibodies: monoclonal phycoerythrin-conjugated anti-CD34 (Milteny Biotec); monoclonal phycoerythrin-conjugated anti-CD13; monoclonal anti-CD90 (BD Biosciences Pharmigen), CD73 (BD Biosciences Pharmigen), CD105 (DAKO) and CD11b (DAKO). Non-conjugated mAbs were stained after washing in 0.1% BSA/PBS with FITC-conjugated anti-mouse IgG (1:1000, Chemicon). After washing, cell fluorescence signals were determined immediately using a FACScan flow cytometer (Becton Dickinson, San Jose, CA, USA) equipped with an argon laser emission of 488 nm. The analysis was performed using CellQuest Software (Becton Dickinson, San Jose, CA, USA).

### Scanning Electron Microscopy (SEM)

Cultured cells were prefixed in a solution containing 1% paraformaldeyde, 1.5% glutaraldehyde in 0.15 M cacodylate buffer for 2 hours at room temperature. After washes with PBS, samples were dehydrated with acetone and critical point dried using carbondioxide. Specimens were then briefly coated with platinum in a Emitek K575-E sputtering apparatus and observed with a Hitachi S4000 FE scanning electron microscope.

## Authors' contributions

FS contributed to the design of the experiments, to the cell culture and flow cytometric analyses; CR and RP contributed the RT-PCR, western blot and immunofluorescence; MB, MAS and FA contributed to taking and isolation of bone marrow stem cells; MSR participated in the design of the study; VS were involved in the writing of the manuscript. All authors read and approved the final manuscript.

## Figures and Tables

**Table 1:** Primer sequences and cycling conditions in RT-PCR

	Lenght of amplified	Primer sequences	Cycles
Nestin	220 bp	5'GCCCTGACCACTCCAGTTTA3' 5'GGAGTCCTGGATTTCCTTCC3'	**33 cycles**: 30 sec, 94°C 30 sec, 55°C 30 sec, 72°C
Vimentin	200 bp	5'GGGACCTCTACGAGGAGGAG3' 5'CGCATTGTCAACATCCTGTC3'	**30 cycles**: 30 sec, 94°C 30 sec, 55°C 30 sec, 72°C
NF-M	333 bp	5'TGGGAAATGGCTCGTCATTT3' 5'CTTCATGGAAGCGGCCACTT3'	**35 cycles**: 30 sec, 94°C 60 sec, 55°C 90 sec, 72°C
NSE	329 bp	5'TGAACACAGACGCTATGCGCTCAG3' 5'CACCTTTATGTGAGTGGACACAGA3'	**30 cycles**: 60 sec, 94°C 60 sec, 60°C 120 sec, 72°C
Hes1	349 bp	5'ACACGACACCGGATAAACCAA3' 5'CGAGTGCGCACCTCGGTA3'	**25 cycles ** 30 sec, 94 °C 30 sec, 60 °C 30 sec, 72 °C
GAPDH	347 bp	5'GCCAAAAGGGTCATCATCTCTG3' 5'CATGCCAGTGAGCTTCCCGT3'	**25 cycles**: 30 sec, 96°C 60 sec, 58°C 30 sec, 74°C
